# Arthroscopically Resected Ganglion Cyst Arising From the Infrapatellar Fat Pad: A Report of Two Cases

**DOI:** 10.7759/cureus.93181

**Published:** 2025-09-25

**Authors:** Ryota Cha, Yuji Arai, Shuji Nakagawa, Kenji Takahashi

**Affiliations:** 1 Orthopaedic Surgery, Japanese Red Cross Kyoto Daiichi Hospital, Kyoto, JPN; 2 Orthopaedics, Kyoto Prefectural University of Medicine, Kyoto, JPN; 3 Sports and Para-Sports Medicine, Kyoto Prefectural University of Medicine, Kyoto, JPN

**Keywords:** anterior knee pain, arthroscopy, ganglion cyst, infrapatellar fat pad, multifocal mass

## Abstract

Most intra-articular ganglion cysts of the knee develop from the cruciate ligament or meniscus and rarely from the infrapatellar fat pad. We report two cases of arthroscopically resected ganglions arising from the infrapatellar fat pad. Case 1: A 24-year-old woman presented with anterior knee pain and a mass. Imaging revealed a diffuse multifocal mass anterior to the knee joint and a mass arising from the infrapatellar fat pad. Resection was performed, with no ganglion recurrence seen six months postoperatively. Case 2: A 61-year-old woman presented with anterior medial knee pain. Imaging revealed medial-type osteoarthritis changes at KL2 and a multifocal mass in the infrapatellar region. Resection plus tibial osteotomy was performed, with no ganglion recurrence observed two years postoperatively. In both cases, arthroscopic resection was performed, and no postoperative recurrence was observed. Arthroscopic resection of ganglions arising from the infrapatellar fat pad may be useful.

## Introduction

Cystic lesions around the knee joint are a common condition encountered in clinical practice. Most cystic lesions around the knee joint are popliteal cysts, while cystic lesions occurring within the knee joint are relatively rare [1]. One type of cystic lesion that develops within the knee joint is a ganglion cyst, but most of these are derived from the cruciate ligament or meniscus, while those originating from the infrapatellar fat pad are relatively rare [2-12]. Additionally, many cysts are incidentally discovered and treated conservatively; however, when a cyst causes pain or joint dysfunction, resection may be necessary, and careful consideration is required for treatment [13]. We report two cases of arthroscopically resected ganglions arising from the infrapatellar fat pad.

## Case presentation

Case 1

**Figure 1:**
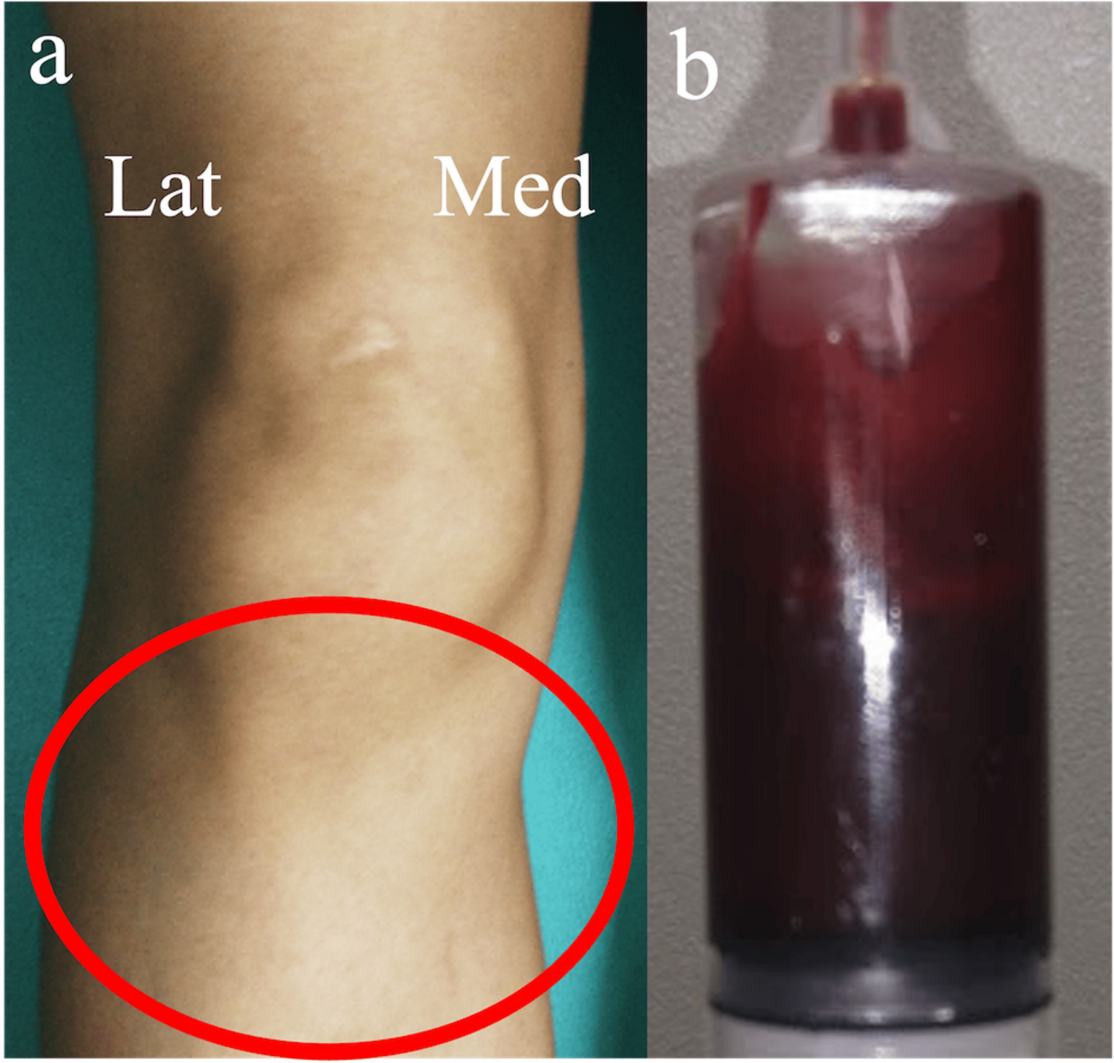
Preoperative clinical images of Case 1 (a) Swollen patellar tendon medially and laterally, (b) The puncture fluid of the mass was red and viscous.

**Figure 2:**
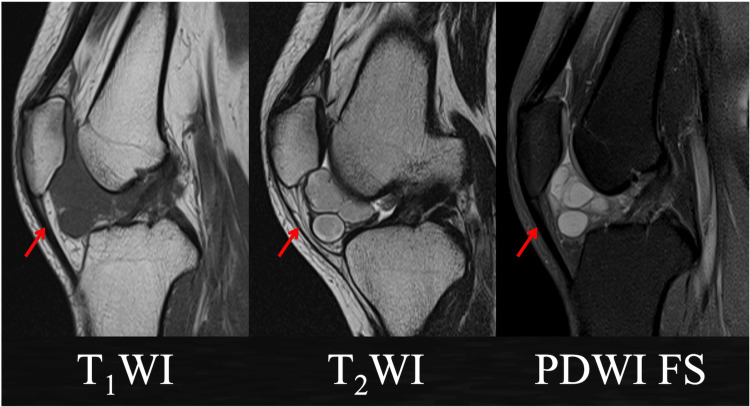
Magnetic resonance images of the knee A multifocal mass in the infrapatellar fat pad was observed, with a low signal on T1WI, a high signal in the periphery, and an equally high signal on T2WI, with no fat suppression and no damage to the articular cartilage, meniscus, or ligaments.

A 24-year-old woman working in garment sales, who had been repeatedly kneeling, presented with anterior right knee pain and a mass. A mass was observed on the medial and lateral sides of the patellar tendon, with associated tenderness in the lateral joint cleft (Figure 1a). The range of motion was 5-145°, with no patellar ballottement or ligamentous instability. The patient was negative for the McMurray test. Imaging showed mild enhancement of the soft tissue shadow just below the right patellar tendon, while magnetic resonance imaging (MRI) revealed a diffuse multifocal mass in the infrapatellar fat pad. The mass had low-signal density on T1WI. On T2WI, the mass had high signal density peripherally, but iso-high signal internally. Fat suppression was not observed. There was no apparent damage to the articular cartilage, meniscus, or ligaments (Figure 2). When the mass was punctured, a highly viscous red-colored effusion was drained (Figure 1b). The patient’s symptoms improved temporarily, but pain was observed later. The diagnosis of an intra-articular ganglion in the right knee joint was made, and the patient was referred for arthroscopic resection.

**Figure 3:**
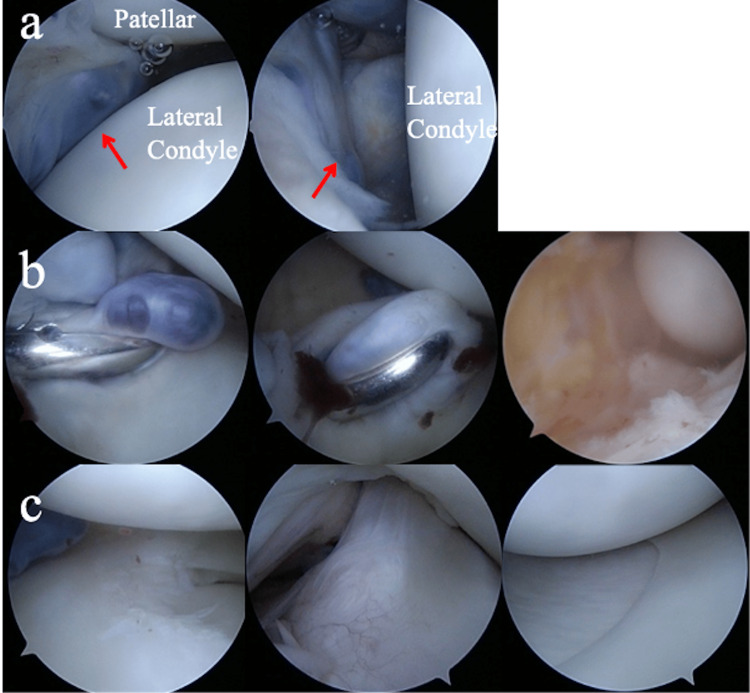
Arthroscopic views (a) Indigo carmine-stained mass arising from the infrapatellar fat pad (red arrow), (b) Red and viscous mass contents. Mass wall was excised, (c) A partial tear of the lateral meniscus was observed. No damage to the surrounding ligaments was observed.

**Figure 4:**
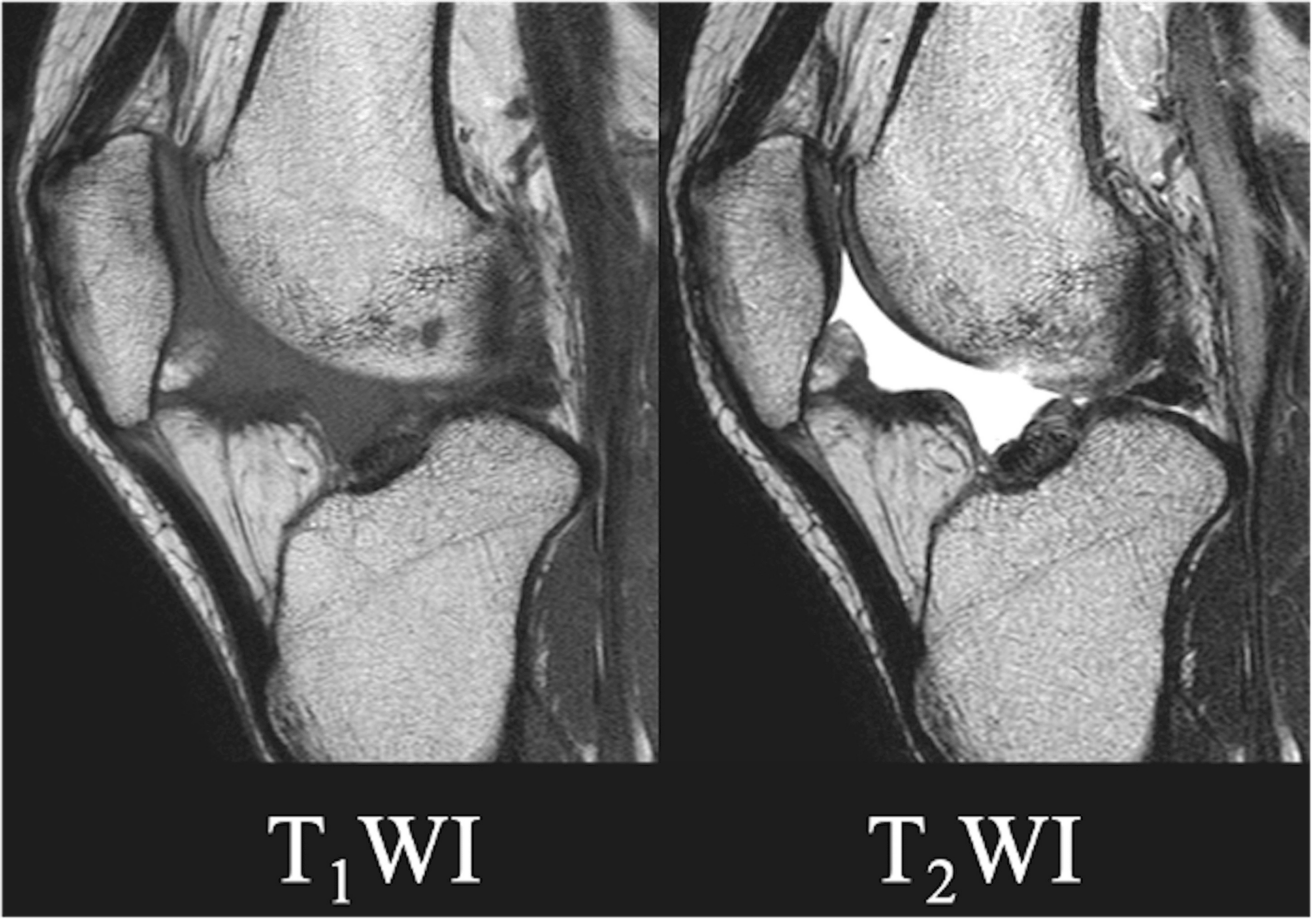
Magnetic resonance images of the knee (six months postoperatively) The ganglionic cyst was no longer visible.

The mass was located laterally, and arthroscopy was initiated through the medial infrapatellar portal. Indigo carmine was injected into the ganglion, whose location was confirmed by palpation, and a pigment-stained mass, arising from the infrapatellar fat pad, was observed anterior to the lateral femorotibial joint (Figure 3a). Since it was challenging to create a lateral sub-patellar portal that avoided the mass, it was necessary to penetrate it. A dark red, highly viscous, hematoma-like fluid, similar to the preoperative puncture fluid, flowed out. A punch biopsy was performed, and the mass wall was excised with a shaver until the blue-stained mass was completely removed, exposing the sublabial fat pad (Figure 3b). A horizontal tear of the lateral meniscus, from the mid to posterior segment, was observed, but there was no communication between the meniscus and the mass. Partial resection of the lateral meniscus was performed. No damage to the medial meniscus, anterior cruciate ligament, posterior cruciate ligament, or articular cartilage was observed (Figure 3c). Pathological examination revealed a thick fibrous cyst wall, lined by synovial surface cells, consistent with a ganglion. The patient’s pain reduced postoperatively, and no ganglion recurrence was observed on an MRI six months postoperatively (Figure 4). The patient was asymptomatic three years later.

Case 2

**Figure 5:**
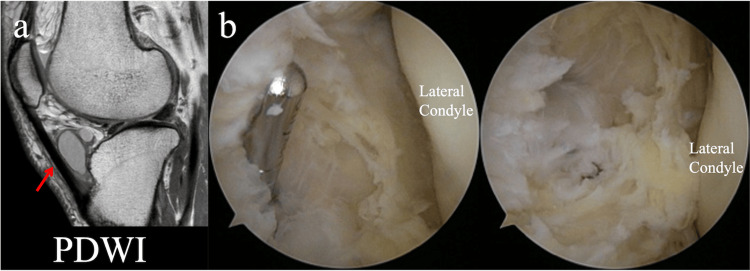
Images of Case 2 (a) Magnetic resonance image of the knee. As in case 1, a multifocal mass was found in the infrapatellar fat pad, (b) Arthroscopic views. As in case 1, the mass wall was excised until the normal sublabial fat pad was exposed.

A 61-year-old woman, a housewife, presented with right knee medial pain and a mass. The mass was observed on the lateral side of the patellar tendon, with associated tenderness in the medial joint cleft. Imaging revealed KL2 medial-type OA changes, and the patient underwent a tibial osteotomy of the right knee. MRI revealed a diffuse mass in the infrapatellar fat pad (Figure 5a). An arthroscopic resection was also planned.

The mass was located laterally, and arthroscopy was initiated through the medial infrapatellar portal. A mass arising from the infrapatellar fat pad was observed anterior to the lateral femorotibial joint. A dark red, highly viscous, hematoma-like fluid flowed out from the creation of a lateral infrapatellar portal. A punch biopsy was performed, and the mass wall was excised with a shaver until the infrapatellar fat pad was exposed (Figure 5b). No disturbance was observed on the meniscus or cruciate ligament. Pathological findings were consistent with a ganglion. Postoperatively, the patient’s pain reduced, and no ganglion recurrence was observed on implant removal after nine months. The Knee Injury and Osteoarthritis Outcome Score (KOOS) score improved from 66.1 points preoperatively to 77.4 points one year postoperatively, and the patient was asymptomatic two years postoperatively.

## Discussion

Ganglions are mass lesions that occur in various locations, such as tendon sheaths, muscles, and periarthrodesis. These occur infrequently in the knee joint (0.2-1.9%) [1]. Ganglions mostly originate from the cruciate ligament or meniscus, and rarely originate from the infrapatellar fat pad. Symptoms of ganglions originating from the infrapatellar fat pad include limited range of motion [2], pain [3,4,11], and swelling [5,6,11], but they are often asymptomatic and discovered incidentally [7,12], as in the second case. Although the exact pathophysiology remains unknown, stray synovial tissue, posttraumatic connective tissue degeneration, mucin degeneration of connective tissue, and proliferation of mesenchymal stem cells have been mentioned as related factors [2,5,6]. In the first case, we considered the cause to be connective tissue degeneration due to repetitive kneeling movements.

Ganglions appear as septate structures with distinct borders on MRI [1]. The structures have clear borders, with low-signal density on T1, high signal density on T2, and sufficient fat suppression [1]. Differential diagnoses include localized pigmented villonodular synovitis, synovial lipoma, synovial haemangioma, and synovial osteochondromatosis [6,7]. Contrast-enhanced MRI may be used to create a definitive diagnosis [2,8,12] and mass localization. However, if the mass is also present in the meniscus, it may be difficult to distinguish its origin, even during arthroscopy. In the present case, there was no communication with the meniscus or ligaments, and the diagnosis of a ganglion arising from the infrapatellar fat pad was made.

Treatment options include puncture aspiration, arthroscopic surgery, and surgical excision through an arthrotomy. Although good results have been observed with puncture aspiration using echocardiography and steroid injection [3,9], there have been a few reports of recurrence [5,6]. In the present case, puncture aspiration was performed; however, recurrence was observed, with no improvement noted. Arthrotomy is often indicated in cases of large masses or extra-articular involvement [2,6,7,11], and there are few reports of recurrence; however, this technique is more invasive than arthroscopic resection. Arthroscopic resection is usually indicated for masses confined to the intra-articular and intra-fatty areas [4], but there have been reports that even large masses extending outside the joint could be resected without recurrence [5]. Additionally, injection of indigo carmine has been reported to enable visualization of the tumor and identification of its borders, making it useful for complete excision of ganglion cysts. [14-16]. In the present cases, arthroscopic surgery was performed since the mass was multifocal and extensive, but was localized within the joint and fat pad. The entire mass could be observed arthroscopically, and indigo carmine was injected into the mass to guide resection. Dissection of all stained mass walls with a shaver allowed dissection until normal fat bodies were observed. Postoperative MRI and a second observation at the time of plate removal showed no recurrence. The patients had a good outcome, suggesting the arthroscopic technique using indigo carmine was successful. This study has the limitation of a short follow-up period, making it impossible to determine long-term effects.

## Conclusions

This report presents two cases of relatively rare ganglions of the infrapatellar fat pad. Arthroscopic resection was performed in both cases, without postoperative recurrence. In cases of multifocal and extensive ganglions localized in the infrapatellar fat pad, arthroscopic resection may be a successful technique.
